# Standing up against office sitting: A study protocol

**DOI:** 10.4102/sajp.v76i1.1415

**Published:** 2020-09-04

**Authors:** Philippe Gradidge, Merling Phaswana, Katrien Wijndaele, Nigel Crowther, Catherine Draper

**Affiliations:** 1 Centre for Exercise Science and Sports Medicine, Faculty of Health Sciences, University of the Witwatersrand, Johannesburg, South Africa; 2 MRC Epidemiology Unit, University of Cambridge School of Clinical Medicine, Cambridge, United Kingdom; 3 Department of Chemical Pathology, National Health Laboratory Service, Faculty of Health Sciences, University of the Witwatersrand, Johannesburg, South Africa; 4 MRC/Wits Developmental Pathways for Health Research Unit, Faculty of Health Sciences, University of the Witwatersrand, Johannesburg, South Africa

**Keywords:** randomised controlled trial, employee wellness, sedentary behaviour, workplace, intervention

## Abstract

**Background:**

Sedentary behaviour is associated with cardiometabolic diseases amongst office-bound workers, mostly through extended sitting and engaging in low-energy-demanding activities during work hours. The aim of this study is to assess the effectiveness of standing desks and healthy messages on cardiovascular parameters in a cohort of office-based workers and to explore the perceptions of these workers about the suitability of this intervention to lower occupation-related sedentariness.

**Methods/design:**

The protocol will use a mixed-methods study design. Phase 1 of this study is a 12-month, single blinded, randomised controlled trial, which will include baseline, 3-month, 6-month and 12-month post-intervention assessments of plausible cardiometabolic risk biomarkers in office-bound workers at a South African credit and information management company. These biomarkers include anthropometry, sedentary behaviour and physical activity, sleep duration, blood pressure, glucose, glycated haemoglobin (HbA1c), lipid profile and cardiorespiratory fitness. Participants will be randomised into an intervention or control group. The intervention group will be provided with an adjustable sit–stand desk and receive weekly health-promoting messages for the intervention period. Phase 2 will use focus group discussions conducted post-intervention to explore the study participants’ perceptions of the effectiveness of the intervention. Cardiometabolic risk biomarkers and changes in these variables will be compared between the intervention group and the control group at the four time points using descriptive and inferential statistics.

**Discussion:**

Regression analysis will be undertaken to determine the association of cardiometabolic risk biomarkers with cardiometabolic diseases. A thematic content analysis approach will be used to explore emerging themes from focus group discussions.

**Protocol identification:**

Pan African Clinical Trial Registry, PACTR201911656014962.

## Introduction

The global impact of preventable cardiometabolic and non-communicable diseases remains excessively high; however, low- to middle-income countries (LMICs) such as South Africa bear the greatest burden (World Health Organization 2018). Insufficient physical activity is still the main behavioural risk factor driving the increase in preventable diseases in South Africa. The demands on employee productivity are high, resulting in prolonged sedentary behaviour, particularly amongst clerical workers (Clemes et al. 2014a). Sedentary behaviour is defined as ‘awake’ activities with limited energy expenditure, while in a sitting, reclining or lying posture (Tremblay et al. 2017). Continuous time in sedentary activities is associated with increased risk of cardiometabolic diseases and inflammatory markers, independent of moderate-to-vigorous physical activity (Hadgraft et al. 2020). Domains of sedentary behaviour include time spent sedentary during occupation, transport and outside of working hours (Clemes et al. 2014b). Workers in office-bound occupations are at an increased risk of being sedentary for most of the occupation-related domain (Chau et al. 2010). A study demonstrated that employed individuals use motorised transport for an average of 0.63 ± 0.68 h/day (Clemes et al. 2014b). This study demonstrated that participants who sat for more than 80% of the workday had higher sitting times during motorised commuting compared with those who sat for < 58% of the workday (1.07 ± 0.98 hours per day vs. 0.35 ± 0.27 hours per day, respectively; *p* < 0.0001) (Clemes et al. 2014b). The adult working population in high-income countries has been found to accumulate sedentary behaviour whilst at work, during recreational time at home and during travel time (Chau et al. 2010). Compounded by sedentary activities outside of work, this accumulated sedentary behaviour may increase the risk for cardiovascular disease, type 2 diabetes and all-cause mortality beyond certain thresholds, even adjusting for physical activity (Patterson et al. 2018). Even in LMICs, worker productivity is becoming increasingly reliant on technology-based solutions with little need to move from the seated position for any tasks, which encourages sedentary behaviours (Gradidge 2017). The implications include limited employee productivity, mental health concerns, financial strain and increased absenteeism. It is therefore important to address sedentary behaviour in the workplace.

A study in a high income country demonstrated reductions in cardiovascular disease risk amongst obese office workers following an intervention to address high sitting time by introducing sit-stand desks rather than the introduction of more physical activity (Healy et al. 2017). This study was a multicomponent intervention, including organisational, environmental (sit–stand desks) and individual components, and significantly better improvements were observed in glycaemic control and insulin resistance in the long term (12 months) compared with the short term (3 months) (Healy et al. 2017). Other studies have shown that environmental interventions such as sit–stand desks, electric height adjustable workstations and active workstations (treadmill and cycle ergometer workstations) result in lower sitting time and improvements in work-related activities (Edwardson et al. 2018; Shrestha et al. 2018). Healthy messages, different from guidelines, help influence individuals into a feasible behaviour pattern (Latimer et al. 2010). The public and occupational health guidelines to lower sedentary time, for instance, are made more acceptable through appealing messaging, such as ‘move more and sit less during the day’ (Holtermann et al. 2020). Evidence shows that workplace interventions to reduce sedentary behaviour can be facilitated by healthy messaging to improve behaviour modification (Healy et al. 2017; Manini et al. 2015).

Commonly, the messages aim to persuade individuals that physical activity is appealing and achievable. These supplementary messages can take many forms and can be disseminated through a variety of messaging processes.

However, there is still a paucity of evidence on the effect of workplace health programmes to reduce sedentary behaviour (Hadgraft et al. 2020). Thus far, only one LMIC intervention study has demonstrated a drop in sedentary behaviour amongst office workers, by using tailored mobile text messages to interrupt sitting time (Dunning et al. 2018). Messages reminding the study participants in the intervention group to stand up and take regular short walks were sent every 20 min during working hours in the working week, for the duration of the 10-week trial. This pilot study did not show a change in anthropometry and cardiometabolic disease markers. Indeed, there is an urgent need to address the cardiovascular disease risk profile of South African workers (Patel et al. 2013), and the place of employment may offer the opportunity to develop programmes to improve worker health and wellness (Patel et al. 2010; World Health Organization 2018).

The aim of this study is to assess the effectiveness of standing desks and healthy messaging (from the authors) on the risk of cardiovascular disease and sedentary behaviour in a cohort of office-based workers and to explore their perceptions on the effectiveness of this intervention to lower sedentariness in the workplace. This manuscript will provide information on the framework used to answer the intended study aims.

## Methods

### Study design, setting, participants and selection

This study will use a mixed-methods study design. Phase 1 of the study is a 12-month single-blind randomised controlled trial (RCT). Phase 2 will explore participant perceptions of the effectiveness of the intervention in the workplace.

In the first phase, the study participants will be assigned to an intervention group for 12 months or a control group. They will be randomised into one of two groups: (1) the combined standing desk–healthy messages group or (2) the control group.

The randomisation will be conducted by a qualified biostatistician independent from the core research team.

The potential for contamination will be minimised by adhering to various evidence-based approaches (Magill et al. 2019): (1) participants will be randomised by business unit, (2) participants will be requested not to share details of the intervention, (3) researchers will be asked not to share details of the intervention and (4) a sufficient number of research assistants will be recruited, each allocated to a single business unit. The RCT will be single blinded so that the first and second authors (assessors) are blinded to the group allocation of the study participants.

All adult office workers (18–65 years) from a specific credit and information management company based in Johannesburg (South Africa), working at least 50% of the workday at their desk, 5 days per week, will be invited to participate by the human resources analyst concerned with employee health and wellness through email and telephonic invitations. The inclusion criteria include access to telephone or mobile, email and/or internet and a desk or workstation within the setting; the ability to communicate in English; and the ability to walk or stand for at least 10 min. The exclusion criteria include participants who are pregnant, non-ambulatory or have a diagnosed medical or chronic condition that affects their ability to stand for 10 min or more. Furthermore, participants with other contraindications for working in the standing position and participants not working at the study site will also be excluded from the study. The total current staff complement of the company is 300, and all employees are housed in the same building. The building has three floors, with each respective floor subdivided securely by business unit, thus restricting the movements of staff in the setting.

The sample size for phase 1 of the study will be determined based on the available resources and the assumption that at least 60 participants per group, adjusted for drop-out, will provide > 90% power to detect a significant difference (*p* < 0.05) in sedentary behaviour between the groups (O’Connell et al. 2015).

For phase 2, semi-structured focus group discussions, conducted by author 1, author 2 and research assistants, are planned with six groups of participants selected by participation in the intervention; each group will have six to eight participants. The principle of saturation in its purest sense (as in grounded theory methodology) will not be applied (Creswell 2007). However, the authors will apply the principle of saturation in the sense that it is anticipated that the sample size will be adequate to provide sufficiently rich data, and that a greater sample size would not yield a significant amount of new information (Tracy 2010).

## Intervention

The combined standing desk–healthy messages group (group 1) will be provided with an adjustable sit–stand desk. The desk is designed for placement on top of the participants’ existing workstation, providing the opportunity to transition from sitting to standing without interrupting productivity. Research assistants, qualified in exercise science, biokinetics or physiotherapy, will set up the participants’ workstations in the most appropriate ergonomic position. The correct configuration will therefore be individualised for each study participant. Participants will be visited weekly by the research assistants to examine the set-up, monitor usage and encourage standing-based work. The participants will be asked to break up extended sitting time by accumulating bouts of standing activity, short intermittent bouts of ≥ 10 min initially and then progressing to longer bouts of ≥ 30 min over the course of the intervention (Smith et al. 2017). Regular communication on the benefits of interrupting sitting will be emailed to the participants. The combined standing desk–healthy messages group will also receive health-promoting messages from the authors focussed on lifestyle behaviour modification through email, short message services and telephonic communication once a week during working hours. The participants in the control group (group 2) will continue using their ‘traditional’ workstations as usual.

## Measures

### Phase 1

Data for the RCT will be collected at baseline, 3 months (short term), 6 months (medium term) and 12 months postintervention (long term) using validated measures and standardised assessments.

#### Body composition

Total body weight (kg) will be measured to the nearest 0.1 kg using a calibrated digital weighing scale (Omron HN-288, Hoofddorp, The Netherlands), and standing height will be measured to the nearest millimetre using a calibrated portable stadiometer (Seca 213, Hamburg, Germany). The participants will wear minimal clothing and will not have shoes on during the measurements. Trained student researchers and author 2 will conduct the measurements. Body mass index (BMI, kg/m^2^) will be calculated and classified as underweight (< 18.5 kg/m^2^), normal weight (≥ 18.5 kg/m^2^ and < 25 kg/m^2^), overweight (≥ 25 kg/m^2^ and < 30 kg/m^2^) or obese (≥ 30 kg/m^2^) (American College of Sports Medicine 2017). Using a flexible, but inelastic, measuring tape, a measurement of the waist circumference will be taken at the narrowest part of the trunk, horizontally, whist the participants are standing with arms at the side, relaxed abdomen and feet together (American College of Sports Medicine 2017). Similarly, the hip circumference measurement will be taken at the widest circumference of the proximal thigh, just under the fold of the gluteus, with feet separated slightly (American College of Sports Medicine 2017). Central obesity is defined as a waist circumference ≥ 80 cm for females or ≥ 94 cm for males (Alberti et al. 2009).

#### Blood pressure

The Omron M7 (Intelli IT [HEM-7322T-E], Kyoto, Japan) will be used to record brachial blood pressure (BP). The device has been validated for determining systolic and diastolic BP (El Feghali et al. 2007). Three measurements will be taken after the participant has rested (≥ 5 min) in the seated position with an appropriately sized cuff around the right upper arm, supported at the level of the heart (American College of Sports Medicine 2017). The average of the last two BP measurements will be recorded. Hypertension will be diagnosed as resting systolic BP ≥ 140 mmHg and/or a resting diastolic BP ≥ 90 mmHg, or taking antihypertensive medication. The undiagnosed participants will be advised to seek medical attention.

#### Blood samples

A finger prick test will be used to collect non-fasting capillary blood samples. Random glucose will be measured with the HemoCue Glucose 201RT system (Ängleholm, Sweden), and random total cholesterol, high-density lipoprotein cholesterol and serum triglycerides will be measured with the CardioChek Plus analyser (Polymer Technology Systems, Inc.). The CardioCheck Plus analyser demonstrates good clinical agreement with a reference analyser, ranging from 95% to 98% (Ferreira et al. 2015). Glycated haemoglobin (HbA1c) will be tested using the HemoCue HbA1c 501 system (Pillay et al. 2019). The HemoCue HbA1c 501 system correlates with laboratory HbA1c tests (rho = 0.995; *p* < 0.001). The HemoCue Glucose 201RT system compares with laboratory methods (coefficient of variances < 6.5%) (Kos et al. 2012; Segerhag et al. 2015). A diagnosis of diabetes is considered as having random plasma glucose of 11.1 mmol/L, HbA1c reading ≥ 6.5% or evidence of diabetic medication. Abnormal total cholesterol will be considered as ≥ 4.5 mmol/L or medication for the management of hypercholesterolemia. Participants undiagnosed for elevated total cholesterol or blood glucose will be advised to seek medical attention.

#### Accelerometry

The small, lightweight and wrist-worn Axivity accelerometer, version AX3 (Newcastle-upon-Tyne, United Kingdom) will be used to collect free-living sleep, sedentary behaviour, light physical activity and moderate-to-vigorous physical activity data. The AX3 has been used in large-scale surveillance studies such as the UK Biobank study (Doherty et al. 2017). This wrist-worn monitor shows excellent agreement with the gold standard doubly labelled water for estimating total energy expenditure (rho = 0.90 and *p* < 0.05 in the dominant wrist; rho = 0.91 and *p* < 0.05 in the non-dominant wrist) (White et al. 2019). The AX3 will be initialised to capture triaxial acceleration data at 100 Hz with a dynamic range of ± 8 g. Participants will be asked to wear the device on their wrist during all hours of the day, over a period of 7 days, except for water-related activities that are not considered as water resistant (e.g. bathing and swimming). Data processing and analysis methods are described elsewhere (Doherty et al. 2017). Data captured by the AX3 will be processed and analysed using an open-source software project developed and used by the UK Biobank Study (https://github.com/activityMonitoring/biobankAccelerometerAnalysis) and the Open Movement AX3 open source software (OmGui version 1.0.0.39, Newcastle University, Newcastle upon Tyne, United Kingdom).

#### Tools

Participants will be asked to complete an appropriate questionnaire to identify self-reported sociodemographic characteristics (age, salary band, position in the company, highest level of education and smoking status), sedentary behaviour, diet and beverage consumption, mental health and absenteeism.

Self-reported sedentary behaviour in the previous 7 days will be quantified using the Last 7 Days Sedentary Time Questionnaire (SIT-Q-7d). The questionnaire includes self-reported sedentary time in five main domains as well as sleep and nap time. The domains include sedentary time during meals, travel, work, recreation and during other non-specified sedentary time. The SIT-Q-7d is acceptable for use in epidemiological studies, with criterion validity for domain-specific variables ranging from 0.22 to 0.76 (Wijndaele et al. 2014).

Habitual consumption of vegetables, fruit, whole grains, dairy, meats and poultry over the past year will be determined using a self-reported dietary intake questionnaire (Kolbe-Alexander et al. 2008). A beverage intake questionnaire (BEVQ-15) quantifies the amount of unsweetened beverages, sugar-sweetened beverages and alcohol consumed by the participants, with validity between 24-h food item recall and BEVQ-15 ranging from rho values of 0.69 to 0.76 (*p* < 0.001) (Hedrick et al. 2012). The estimated number of kilocalories per drink will be calculated.

The Centre for Epidemiological Studies Depression Scale (CES-D-10) is a validated questionnaire (rho = 0.81–0.94, *p* < 0.05 [Baron, Davies & Lund 2017]) that estimates the mental health status of employees, specifically eliciting symptoms of depression in the past 7 days (Andresen et al. 1994). The tool includes items on the effect of depression, somatic symptoms and positive effects. A four-point Likert scale is used for each item, ranging from ‘rarely or none of the time’ to ‘all of the time’.

#### Cardiorespiratory fitness

The Queens College 3-min step test will be used to estimate cardiorespiratory fitness (using maximal oxygen uptake [VO_2max_]). The agreement between this step test and measured VO_2max_ is high (rho = 0.75, *p* < 0.001). The step test uses a 41.3-cm step with a stepping rate of 24 steps/min for men and 22 steps/min for women for a period of 3 min (American College of Sports Medicine 2017). Within 5 s of the participant sitting, a research assistant will measure the post-exercise heart rate (HR). Maximal oxygen uptake (VO_2max_) will be calculated for men (VO_2max_ = 111.33 – [0.42 × HR]) and women (VO_2max_= 65.81 – [0.1847 × HR]).

### Phase 2

All study participants involved in the combined standing desk–healthy messages intervention and who did not drop out of the RCT will be invited to participate in semi-structured focus groups at intervention follow-up to evaluate their perceptions of the intervention – receiving the healthy messages and/or using the standing desks – and their perceptions of the effectiveness of using these interventions in the workplace. Six focus groups are planned, with six to eight participants per group. Given that this intervention group will have approximately 60 participants, and that not all participants will make themselves available for a focus group, the intention to invite all intervention group participants to take part in a focus group accounts for at least a 60% positive response rate. Although the challenges associated with recruitment for focus groups can sometimes make it difficult to stratify focus groups, an attempt will be made to stratify these focus groups based on participants’ responses to phase 1. During the focus group discussions, participants will be asked to comment to facilitators and address extended sitting time whilst working, and to discuss the most suitable methods to improve health in their place of work. The focus groups will be approximately 45 – 60 min in duration.

For this qualitative component of the project, the following ‘big-tent’ criteria of qualitative quality will be considered and applied: a worthy topic, rich rigour, sincerity, credibility, resonance, significant contribution, ethical and meaningful coherence (Tracy & Hinrichs 2017). At this stage of the study, it can be argued that this study addresses a worthy topic (the prevention of non-communicable diseases (NCDs) in an LMIC) and could make a significant contribution to the literature on this topic in South Africa. Furthermore, in relation to rich rigour, appropriate sampling, data collection and data analysis methods have been planned; ethical considerations are discussed below. Sincerity, credibility, resonance and meaningful coherence are criteria that are not really possible to apply at this stage of the research, given that this is a protocol paper and does not yet report on study findings. These criteria are particularly relevant for the interpretation of findings and how these findings are presented.

## Data analysis

All data for the RCT will be analysed using Stata Special Edition (SE) (version 14, Stata Corp, United States of America). Descriptive statistics will be used for comparing outcome data at baseline and change from baseline to follow-up between the intervention and control groups. Paired *t*-tests for within-group comparisons over time or their non-parametric equivalents depending on data distribution will be utilised. Comparison between groups will be made using analysis of variance or the non-parametric equivalent. Multivariable linear regression models will be used to determine if any of baseline study variables modulate these effects on the outcome variables (change in BMI, lipids, BP, HbA1c, VO_2max_ and sedentary behaviour). Significance will be accepted at an alpha level of *p* ≤ 0.05. The Consolidated Criteria for Reporting Qualitative Research (COREq) checklist will be used for reporting on the methodology, research team, study design and analysis, and study findings (Tong et al. 2007).

## Ethical consideration

The study has received ethical approval from the Human Research Ethics Committee, University of the Witwatersrand (ethics certificate number M190224), as well as relevant authorities in the company, and is registered as a trial with the Pan African Clinical Trial Registry (PACTR201911656014962). The South African Medical Research Council has reviewed and awarded a Self-Initiated Research Grant for the study. Participants will be asked to sign informed consent before participating in the RCT. The study participants will be required to give written consent prior to participation in the focus group discussions. In addition, written consent will be sought from the study participants for the recording of the focus group discussions. The study participants will be reminded that participation in the study is voluntary and that the freedom to withdraw will not result in negative consequences.

## Discussion

Workers in office occupations are at increased risk of cardiovascular disease because of prolonged sedentary behaviour (Wolf et al. 2018). One approach to health promotion for the employed population is to address sitting in the workplace. A review of interventions shows that cardiovascular health can be improved through interventions that reduce sedentary behaviour (Dunstan et al. 2012; Edwardson et al. 2018; Hadgraft et al. 2020). The findings of this study will add to a limited body of evidence on addressing sedentary behaviour in the workplace and will be used to inform health-promoting policies and to develop models for disease prevention in the sub-Saharan African workforce. Furthermore, by addressing cardiovascular disease risk in the workplace, overall work-related job performance, productivity and absenteeism may be improved.

## Limitations

The study will be conducted in one setting, and although the study population is diverse in terms of gender, age and socio-economic status, the external validity may be limited.

## Conclusion

In conclusion, this study will provide formative data on understanding sedentariness and the prevention of cardiovascular disease risk factors amongst workers in office-based occupations for further investigation.
